# Potential Regulatory Role in Mammalian Host Adaptation for a Small Intergenic Region of Lp17 in the Lyme Disease Spirochete

**DOI:** 10.3389/fcimb.2022.892220

**Published:** 2022-05-02

**Authors:** Michael A. Crowley, Troy Bankhead

**Affiliations:** Department of Veterinary Microbiology and Pathology, Washington State University, Pullman, WA, United States

**Keywords:** Lyme disease, *Borrelia burgdorferi*, gene regulation, host adaptation, sRNA (small RNA)

## Abstract

The bacterial agent of Lyme disease, *Borrelia burgdorferi*, relies on an intricate gene regulatory network to transit between the disparate *Ixodes* tick vector and mammalian host environments. We recently reported that a *B. burgdorferi* mutant lacking a transcriptionally active intergenic region of lp17 displayed attenuated murine tissue colonization and pathogenesis due to altered expression of multiple antigens. In this study, a more detailed characterization of the putative regulatory factor encoded by the intergenic region was pursued. *In cis* complemented strains featuring mutations aimed at eliminating potential protein translation were capable of full tissue colonization, suggesting that the functional product encoded by the intergenic region is not a protein as previously predicted. *In trans* complementation of the intergenic region resulted in elevated transcription of the sequence compared to wild type and was found to completely abolish infectivity in both immunocompetent "and immunodeficient mice. Quantitative analysis of transcription of the intergenic region by wild-type *B. burgdorferi* showed it to be highly induced during murine infection relative to *in vitro* culture. Lastly, targeted deletion of this intergenic region resulted in significant changes to the transcriptome, including genes with potential roles in transmission and host adaptation. The findings reported herein strongly suggest that this segment of lp17 serves a potentially critical role in the regulation of genes required for adaptation and persistence of the pathogen in a mammalian host.

## Introduction

Lyme borreliosis is an emerging disease with no reliable vaccine that affects an estimated 476,000 people each year in the United States (Kugeler et al., 2021). Unmitigated infection with the causative spirochetal bacterium, *Borrelia burgdorferi*, can result in debilitating clinical manifestations in humans including arthritis, carditis, and neurological disorders (Steere et al., 1987; Steere et al., 1994; Steere and Glickstein, 2004; Bratton et al., 2008). The pathogen is transmitted to humans and other susceptible animals through feeding by infected *Ixodes* ticks.

During transmission from the tick vector to a mammalian host, *B. burgdorferi* undergoes a drastic shift in gene expression in response to a biophysiochemical disparity between the two environments. This shift in gene expression is controlled in part by the Rrp2-RpoN-RpoS two-component regulatory system and has been shown to be triggered by changes in temperature, pH, CO_2_ concentration, host immune pressures, and nutrient availability (Yang et al., 2000; Hubner, 2001; Caimano et al., 2004; Yang et al., 2005; Burtnick, 2007; Caimano et al., 2007; Caimano et al., 2007; Hyde et al., 2007; Caimano, 2011; Dunham-Ems et al., 2012; Radolf et al., 2012; Caimano et al., 2019). When expressed, the RpoS alternative sigma factor promotes expression of a subset of genes that facilitate spirochete transmission and survival in mammalian hosts including those that encode surface lipoproteins required for host interaction such as complement binding proteins and extracellular matrix binding proteins, proteins facilitating evasion of innate and adaptive immune responses, and metabolic genes required to sustain the bacterium’s auxotrophic nature in the host (Kraiczy, 2003; Purser, 2003; Kraiczy, 2004; Fischer et al., 2006; Hartmann, 2006; McDowell, 2006; Seshu, 2006; Tilly et al., 2006; Xu et al., 2006; Bankhead and Chaconas, 2007; Tilly et al., 2007; Siegel, 2010; Carrasco et al., 2015; Caine et al., 2017; Stevenson and Seshu, 2018). Importantly, activation of the RpoS regulon also results in the repression of tick- and transmission-phase associated genes which, if left unrepressed, can result in attenuated ability of spirochetes to establish persistent murine infection (Grove et al., 2017).

To date, study of the transcriptomic shift resulting from RpoS activation has been primarily focused on two aspects: 1) the bacterial, vector, and host factors that induce gene expression changes, and 2) the end effects of these changes on the transcriptome and proteome of the bacterium under various conditions. Intermediate molecular factors bridging Rrp2-RpoN-RpoS activation and its downstream effects are not as well characterized. Attempts to bridge this knowledge gap have turned in part toward the investigation of regulatory small non-coding RNA (sRNA) activities in this emerging pathogen.

It was formerly posited that the *B. burgdorferi* genome encoded only a few sRNA molecules, and lacked any orthologues of sRNA-associated proteins such as the RNA chaperone protein Hfq (Ostberg et al., 2004). However, a series of more recent studies have highlighted the importance of sRNAs in the Lyme disease pathogen as robust facilitators of genetic regulation [Reviewed in (Lybecker and Samuels, 2017)]. Lybecker and Samuels demonstrated that *B. burgdorferi* encodes an Hfq orthologue (Hfq_Bb_) that is required for the function of DsrA, an sRNA important for the translation of RpoS in a temperature-dependent manner (Lybecker and Samuels, 2007; Lybecker et al., 2010). More recently, Medina-Pérez et al. described *ittA*, an lp17-encoded sRNA with an important regulatory role during murine infection (Medina-Pérez et al., 2020).

A study by Poptisch et al. probed the temperature-dependent sRNA transcriptome of *B. burgdorferi* and detected a number of sRNAs that are up-regulated at 37°C compared to room temperature *in vitro*, which is suggestive of a role in mammalian infection (Popitsch et al., 2017). Similar work analyzed the stringent response-regulated sRNA transcriptome using a *relB* mutant clone (Drecktrah et al., 2018). In both of these studies, an lp17-encoded sRNA was detected and denoted SR0726. This respective RNA transcript mapped to an intergenic space that was previously annotated as a predicted ORF, *bbd07.* An RNA transcript from this same region was also detected in a pair of earlier studies investigating Rrp2-, RpoN-, and RpoS-dependent genes, where its expression was shown to be highly dependent on an intact alternative sigma factor pathway (Boardman, 2008; Ouyang et al., 2008). We recently demonstrated that an lp17 left-end deletion mutant lacking this region displays attenuated murine tissue colonization and pathogenicity, which was ultimately attributed to a 317 bp intergenic region encompassing the *bbd07* locus, suggesting that the gene product responsible for the mutant phenotype could be the SR0726 sRNA (Casselli et al., 2019). The tissue colonization defect was not observed during infection of SCID mice, indicating a potential role for this locus in avoidance of adaptive immunity. Further investigation provided evidence that deletion of this region results in dysregulated antigen expression, implicating the gene product as a regulatory factor.

In the current study, we aimed to test the hypothesis that the *bbd07* intergenic region of lp17 encodes for the sRNA SR0726, which participates in the transcriptome and proteome shift required for spirochetes to adapt to the mammalian host environment. To do this, the previously described lp17 left-end mutant was complemented with non-native *bbd07* to rule out the possibility that the phenotype of the mutant results from absence of BBD07 protein production (Casselli et al., 2019). Then, the effects of SR0726 overexpression were studied in the context of murine infection and antigen expression. These tests revealed that spirochetes transcribing high levels of SR0726 cannot infect mice, and that this phenotype may be independent of altered *in vitro* antigen expression. Next, the transcription of SR0726 was quantified under *in vivo* conditions, which further supported its putative involvement in the mammalian portion of the enzootic cycle. Finally, a clone featuring a targeted knockout of the *bbd07* intergenic region was generated and used for RNA-seq analysis, which revealed that SR0726 activity has affects on transcript levels in addition to the previously observed effects on the *in vivo*-expressed antigenic proteome. This work represents a significant step forward toward understanding the critical role that this lp17 intergenic region has in host adaptation by the Lyme disease pathogen.

## Materials and Methods

### Bacterial Strains and Culture Conditions

The *B. burgdorferi* isolate B31-5A4 used in this work has been characterized in previous studies ( (Casselli et al., 2012; Casselli et al., 2019); **Table 1**). *B. burgdorferi* clones were grown at 35°C under 1.5% CO_2_ in modified Barbour-Stoenner-Kelly medium (BSK-II) supplemented with 6% rabbit serum (Cedarlane) (Barbour, 1984). Mutant strains were grown in BSK-II supplemented with kanamycin (200 µg/ml) and/or gentamicin (100 µg/ml) as indicated. Culture density was monitored by visualization under dark-field microscopy using a Petroff-Hausser counting chamber.

**Table 1:** *B. burgdorferi* strains used in this study.

Strain^a^	Description	Reference
**Wild type**	*B. burgdorferi* strain B31 clone 5A4	(Purser and Norris, 2000)
**Δ1-7**	5A4 lacking genes *bbd01-bbd07* (bp 1-4680) from lp17.	(Casselli et al., 2019)
**Comp7N*_c_***	Δ1-7 complemented *in cis* with *bbd07* (*bbd07*/SR0726, bp 4257-4573) sequence of lp17.	(Casselli et al., 2019)
**Comp7stop*_c_***	Δ1-7 complemented *in cis* with *bbd07*/SR0726 with C-A mutation corresponding to bp 4306 of lp17.	This Study
**Comp7NΔstart*_c_***	Δ1-7 complemented *in cis* with *bbd07*/SR0726 with T-C mutation corresponding to bp 4372 of lp17.	This Study
**Comp7N*_t_***	Δ1-7 complemented *in trans* with the Comp7N sequence harbored on pBSV2G.	This Study, (Elias et al., 2003; Tilly et al., 2006)
**Comp7Nstop*_t_***	Δ1-7 complemented *in trans* with the Comp7Nstop sequence harbored on pBSV2G.	This Study, (Elias et al., 2003; Tilly et al., 2006)
**CompE*_t_***	Δ1-7 transformed with empty pBSV2G	This Study, (Elias et al., 2003; Tilly et al., 2006)
**Δ*d07***	5A4 lacking *bbd07* (bp 4257-4573), and cp32-6	This Study
**Δ*d07*Comp*_c_***	Δ*d07* complemented with lp17 lacking *bbd01-bbd03*, and carrying a kanamycin resistance cassette.	This Study, (Casselli et al., 2019)

a Subscripted c or t in complement strain names indicates in cis or in trans complementation, respectively.

### Mutant and Complement Strain Generation and Screening

A list of mutant strains and their corresponding genotypes are provided in **Table 1**. All transformations were performed using electrocompetent *B. burgdorferi* cells as previously described (Samuels, 1995; Bankhead and Chaconas, 2007). Transformations were recovered in drug-free media for 24 hours and plated by limiting dilution with antibiotic selection to isolate clonal transformants. Positive clones were identified by PCR for introduced sequence. *In trans* complement strains were further verified by visualization of the respective shuttle vectors on agarose gel. Endogenous plasmid content was analyzed in all strains by multiplex PCR using primers specific for regions unique to each plasmid as previously described (Bunikis et al., 2011).

### Ethics Statement

The experiments on mice were carried out according to the protocols and guidelines approved by American Association for Accreditation of Laboratory Animal Care (AAALAC) and by the Office of Campus Veterinarian at Washington State University (Animal Welfare Assurance A3485-01 and USDA registration number 91-R-002). These guidelines are in compliance with the U.S. Public Health Service Policy on Humane Care and Use of Laboratory Animals. The animals were housed and maintained in an AAALAC-accredited facility at Washington State University, Pullman, WA. The Washington State University Institutional Animal Care and Use Committee approved the experimental procedures carried out during the current studies.

### Infection, Recovery, and Quantification of *B. burgdorferi* From Mice

Four-week-old male immunocompetent C3H/HeJ (C3H) and immunodeficient C3SnSmn.CB17-*Prkdc^scid^*/J (SCID) (Jackson, Bar Harbor, ME) mice were infected by subcutaneous needle inoculation (near the base of the tail, to the right of the midline) with 100 µl BSK-II containing 5X10^3^ total *B. burgdorferi* cells unless indicated otherwise.

Blood (day 7 post infection), ear (day 14, 21, and 28 post infection), joint, heart, and bladder (day 28 post infection) tissues were collected and cultured in BSK-II supplemented with 20 μg/ml phosphomycin, 50 μg/ml rifampicin, and 2.5 μg/ml amphotericin-B. Dark-field microscopy was used to determine the presence or absence of viable spirochetes for each cultured tissue sample. A sample was deemed negative if no spirochetes could be seen in 10 fields of view after 3 weeks of culture.

For bacterial burden quantification, heart, bladder, joint, and ear tissues were harvested at day 28 post infection and immediately snap-frozen in liquid nitrogen prior to storage at -80°C. DNA was extracted using DNeasy Blood and Tissue Kit (Qiagen, Valencia, CA) following the manufacturer’s instructions. DNA samples were then cleaned and concentrated using Genomic DNA Clean & Concentrator Kit (Zymo Research, Orange, CA). Quantitative PCR for the *B. burgdorferi flaB* and mouse *β-actin* genes was performed in duplex on each sample in triplicate using TaqMan probes and the droplet digital PCR system (ddPCR™, BioRad, Carlsbad, CA). All steps, including droplet generation, thermocycling, and droplet reading were performed following the manufacturer’s instructions. All reactions were performed with 4ng of template DNA. Primers and probes used for this assay are listed in **Table 1**.

### qRT-PCR Analysis

For RNA extraction from *in vitro* cultures, triplicate cultures of wild-type, Δ1-7, Comp7N_c,_ and Comp7N_t_ were grown to late log phase (1x10^8^ spirochetes ml^-1^), pooled, and pelleted by centrifugation. RNA was extracted using a hot phenol method described previously (Jahn et al., 2008; Drecktrah et al., 2015; Popitsch et al., 2017; Drecktrah et al., 2018). For RNA isolation from mouse tissues, three 4- to 6-week-old C3H mice were infected with a total of 10^5^ wild type spirochetes. Infection was verified and monitored as described above. At day 21 post infection, heart, bladder, and joint tissue were harvested and snap frozen in liquid nitrogen. Tissues were homogenized by mortar and pestle under liquid nitrogen. Homogenates were suspended in Trizol reagent (Invitrogen), and RNA was extracted following manufacturer’s instructions using chloroform followed by alcohol precipitation.

Genomic DNA was removed from RNA samples using the Turbo DNase free kit (Invitrogen). cDNA was synthesized from 1μg of RNA using the iScript cDNA synthesis kit (BioRad). Non-quantitative control PCR reactions were performed using RNA and genomic DNA templates corresponding to each sample to verify successful removal of contaminant DNA, primer specificity, and the absence of *bbd07*/SR0726 amplification from Δ1-7 derived DNA. A total of 1uL of cDNA from each sample was diluted 1:10 and added to quadruplicate PCR reactions. PCR was performed using Evagreen supermix and the QX200 ddPCR system according to the manufacturer’s instructions (BioRad). Replicate reactions for each sample were performed at least in quadruplicate.

### Western Blot Analysis

Cultures of indicated strains were grown in triplicate to late log-phase (1x10^8^ cells mL^-1^) under standard culture conditions as described above. Replicate cultures were pooled and pelleted by centrifugation, washed, and resuspended in 90μL PBS. Lysis was performed by adding 30μL of 4X Laemmli loading dye containing 10% β-mercaptoethanol, and then heating the samples to 80°C for 10 minutes following at least one freeze-thaw cycle. Lysates (10^9^ cells) were electrophoresed on a precast 4%-15% polyacrylamide gradient gel (Bio-Rad). Western blotting was performed as described previously (Casselli et al., 2012), using sera harvested from C3H mice infected with 5x10^3^ spirochetes of the indicated strain for 28 days. Purified polyclonal antibodies (Rockland) were used in and anti-FlaB Western blots.

### RNA-Seq

Cultures were grown in triplicate under standard culture conditions to mid-log phase (5x10^7^ spirochetes mL^-1^), then temperature shifted by 1:100 dilution into room temperature BSK-II. Cultures were allowed to grow back to mid log phase, at which point they were subcultured again 1:100 into warm BSK-II. These final cultures were then allowed to grow under standard conditions to late log-phase (1x10^8^ cells mL^-1^). Cultures were pooled, and RNA was extracted using a hot phenol method described previously (Jahn et al., 2008; Drecktrah et al., 2015; Popitsch et al., 2017; Drecktrah et al., 2018). After DNase treatment, RNA was assessed for integrity using an Agilent 2100 Bioanalyzer. Clean, high quality RNA samples were shipped to the Novogene Corporation for library preparation, sequencing, and data analysis. 3 μg RNA per sample was used as input material for the RNA sample preparations. Sequencing libraries were generated using NEBNext^®^ Ultra™ Directional RNA Library Prep Kit for Illumina^®^ (NEB, USA) following manufacturer’s recommendations and index codes were added to attribute sequences to each sample. Following rRNA removal, fragmentation was carried out using divalent cations under elevated temperature in NEBNext First Strand Synthesis Reaction Buffer (5X). First strand cDNA was synthesized using random hexamer primer and M-MuLV Reverse Transcriptase (RNaseH-). Second strand cDNA synthesis was subsequently performed using DNA Polymerase I and RNase H. In the reaction buffer, dNTPs with dTTP were replaced by dUTP. After adenylation of 3’ ends of cDNA fragments, NEBNext Adaptor with hairpin loop structure were ligated to prepare for hybridization. In order to select cDNA fragments of preferentially 150~200 bp in length, the library fragments were purified with AMPure XP system (Beckman Coulter, Beverly, USA). Then 3 μl USER Enzyme (NEB, USA) was used with size-selected, adaptor-ligated cDNA at 37°C for 15 min followed by 5 min at 95°C before PCR. Then PCR was performed with Phusion High-Fidelity DNA polymerase, Universal PCR primers and Index (X) Primer. Lastly, products were purified (AMPure XP system) and library quality was assessed on the Agilent Bioanalyzer 2100 system. The clustering of the index-coded samples was performed on a cBot Cluster Generation System using HiSeq PE Cluster Kit cBot-HS (Illumina). After cluster generation, the library preparations were sequenced on an Illumina platform and paired-end reads were generated. Raw data of fastq format was firstly processed through in-house perl scripts. In this step, clean reads were obtained by removing reads containing adapter, reads containing ploy-N, and low-quality reads. Alignment of clean reads to reference genome was performed with used Bowtie2-2.2.3. (Langmead and Salzberg, 2012). HTSeq v0.6.1 was used to count the read numbers mapped to each gene. Prior to differential gene expression analysis, for each sequenced library, the read counts were adjusted by edgeR program package through one scaling normalized factor. Differential expression analysis between the strains was performed using the DEGSeq R package (1.20.0). P values were adjusted using the Benjamini & Hochberg method (Green and Diggle, 2007).

### Northern Blot Analysis

RNA was extracted from triplicate cultures of indicated strains exactly as described above for RNAseq. Fifteen µg (SR0726) or 1.5 µg (5S) of RNA from each strain was separated using the SequaGel UreaGel 19:1 Denaturing Gel System (National Diagnostics) and transferred to nylon membranes (Rosche) using a Trans-Blot^®^ Turbo^™^ transfer system (BioRad) run at 25V for 45 minutes. After transfer and UV crosslinking, dried membranes were prehybridized with probe-free UltraHyb^®^ oligo hybridization buffer (Invitrogen) for 2 hours at 42°C, then hybridized overnight at the same temperature with biotinylated probe P1339 or P1349 (**Table S1**) at a final concentration of 200 pM. After washing (2X SSC, 0.1% SDS) and blocking (1X Intercept^®^ PBS blocking buffer, 0.5% SDS), membranes were treated with IRDye^®^ 800CW Streptavidin (LI-COR Biosciences) in blocking buffer, then washed with PBST. Detection was performed on the Odyssey^®^ CLx infared imaging system (LI-COR Biosciences).

### Statistical Analyses

Fisher’s exact test was used to determine significant differences in the ability to recover a given recombinant strain by culturing of tissues compared with wild type *B. burgdorferi*. Student’s t-test was performed to determine significant differences in spirochete burden in heart tissue samples from qPCR analyses, where the average burden in heart tissues from mice infected with a given recombinant strain was compared to that of mice infected with the wild type. Student’s t-test was also used to determine significant differences in the average *in vitro* SR0726 transcription levels between a given recombinant strain and the wild type by qRT-PCR. One‐way ANOVA followed by all pairwise multiple comparison (Holm‐Sidak) was used to determine significantly different levels of average *in vivo* SR0726 transcription by the wild type between each tissue tested, as well as significantly different *bb0360 and bb0733* transcription levels between strains by qRT-PCR. All statistical tests were performed using SigmaPlot.

## Results

### Mutations That Disrupt Potential BBD07 Protein Production Do Not Perturb the Functionality of the Gene Product During Murine Infection

The recent detection of a putative sRNA encoded within the intergenic space of lp17 containing the discontinued *bbd07* ORF annotation (NC_001849.1 [discontinued]), coupled with numerous failed attempts by our laboratory to detect a BBD07 protein product, provided strong indication that the functional product encoded by the *bbd07* locus is the SR0726 sRNA transcript (Popitsch et al., 2017).

**Figure 1:**
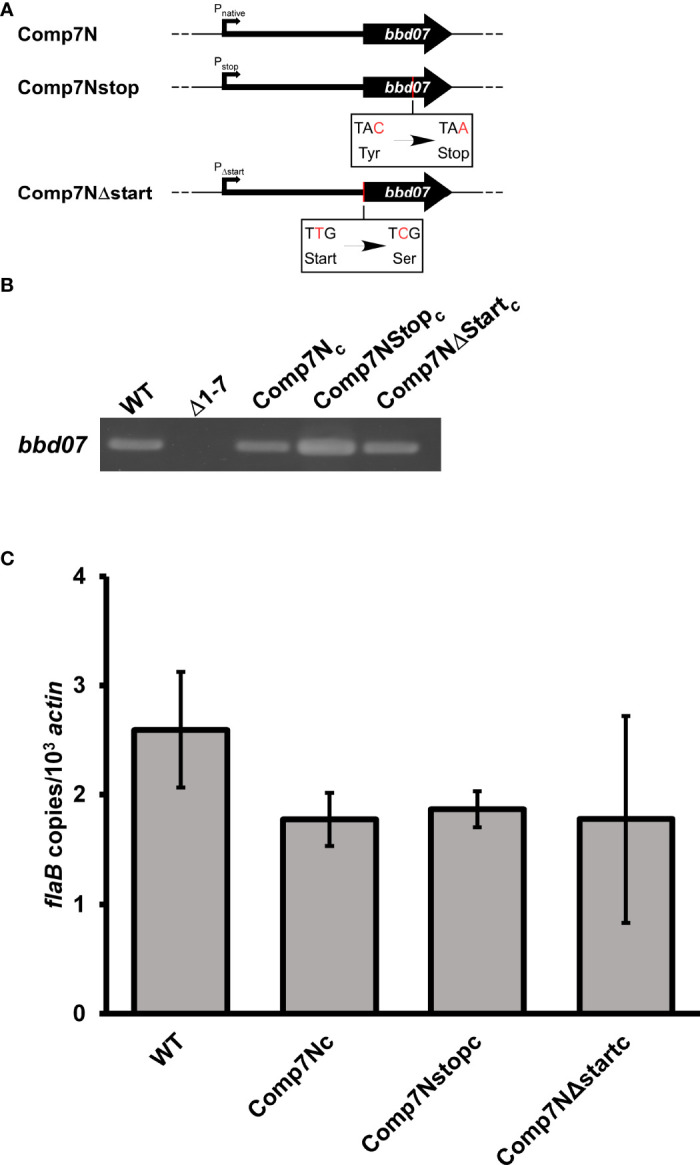
Neither introduction of a stop codon nor disruption of potential start codon abrogates the function of the *bbd07*-encoded gene product. **(A)** Diagrams indicating where mutations were made with respect to the discontinued *bbd07* ORF annotation (thick black arrow, 117 bp). Thick black line upstream of arrow represents 200 bp of upstream sequence included in the constructs. Mutated base pairs are indicated with red text. **(B)** PCR verifications of successful complementation using P1050/P1051. **(C)** Mice were inoculated subcutaneously with indicated strains at a dose of 5x10^3^ total spirochetes. qPCR analysis was used to assess bacterial burden in heart tissues harvested from mice 28 days post-inoculation. Reactions were performed in duplex with probes/primers for *flaB* (P199, P200, P201) and mouse *actin* (P202, P203, P1086). Data bars indicate the average number of *flaB* copies per 10^3^ *actin* copies in heart tissues from 5 mice. Students t-test was performed to assess significance of differences in heart tissue colonization between each recombinant strain and wild type (*P* < 0.05). No significant differences were observed. Error bars indicate SEM for each group (n = 5).

To further rule out the possibility of functional BBD07 protein production, two *in cis* complement strains were generated in our previously described lp17 mutant strain lacking a region containing *bbd01*-*bbd07* that is incapable of murine heart tissue colonization (Δ1-7; (Casselli et al., 2019)). These two genetically complemented strains harbored a *bbd07* copy containing either a premature stop codon or a disrupted start codon, and were denoted Comp7Nstop*_c_* and Comp7NΔstart*_c_*, respectively (**Table 1**). Both constructs included 200 bp of native sequence upstream of the previously annotated *bbd07* that are also present in the previously described native complement Comp7N*_c_* (Casselli et al., 2019). The rationale was that if the genetic region encodes a protein that contributes to the mutant phenotype, then these strains would not be capable of translating the functional protein, leading to a defective tissue colonization phenotype comparable to the Δ1-7 mutant strain. Alternatively, if the functional product encoded by the region is sRNA SR0726, then these mutations would be expected to have no effect on functionality, and thus the strains would exhibit tissue colonization levels comparable to wild-type control and native *cis* complement (Comp7N*_c_*) strains. Single nucleotide mutations were made in frame as defined by the previously predicted TTG start codon at bp 4373 of *B. burgdorferi* B31A lp17(NC_001849). For Comp7Nstop*_c_*, the stop codon was generated *via* a cytosine to adenine mutation corresponding to bp 4306 of the annotated lp17 DNA sequence. For Comp7NΔstart*_c_*, a thymine to cytosine mutation was introduced in the middle nucleotide of the previously predicted start codon that corresponds to bp 4372 of the annotated lp17 DNA sequence (**Figure 1A**). Neither mutation was predicted to significantly perturb the putative RNA secondary structure that might be important for the function of the SR0726 sRNA product. To generate the clones, suicide vector-derived plasmids harboring the 317 bp sequences were used to transform Δ1-7 *B. burgdorferi* cells, and transformants were screened for insertion of the altered sequences in lp17. PCR verification of these clones demonstrated successful complementation as shown in **Figure 1B**. Sequencing was performed to verify the single base pair mutations in each construct, and isogenicity to the parent clone was confirmed by multiplex PCR for native plasmid content (data not shown) (Bunikis et al., 2011).

To determine if these altered sequences were able to rescue the mutant phenotype, the capacity for heart tissue colonization of immunocompetent mice was selected for use as a readout. We reasoned that this was a reliable indicator of gene product functionality due to the fact that the Δ1-7 mutant has been previously shown to be unable to colonize heart tissue in immunocompetent mice (Casselli et al., 2019). Groups of five C3H mice each were needle inoculated (5x10^3^ total spirochetes) with either wild type or one of the various complement clones. Successful establishment of infection was monitored weekly by culturing blood and ear tissues to visually verify the presence of spirochetes by dark field microscopy (**Table 2**). All mice in the experiment displayed spirochetemia at 7 days post infection and remained infected for the duration of the 28-day experiment. Recovery of Δ1-7 spirochetes from ear tissue samples on day 14 post infection was variable in a previous experiment, while here all ear tissue samples taken at this time point from Comp7Nstop*_c_*- and Comp7NΔstart*_c_*-infected mice yielded spirochete growth in culture. At day 28 post infection, heart tissue was collected from each mouse and divided in half longitudinally. One half of each heart was deposited into culture media, while the other half was snap-frozen for DNA extraction and qPCR to determine bacterial burden.

**Table 2:** Heart colonization by non-native *bbd07* complement strains.

	Timepoint/tissue			
Strain	Day 7/blood	Day14/ear	Day 21/ear	Day 28/heart
**WT**	5/5	5/5	5/5	5/5
**Δ1-7**	13/13	ND	ND	0/13*
**Comp7N*_c_***	5/5	5/5	5/5	5/5
**Comp7Nstop*_c_***	5/5	5/5	5/5	5/5
**Comp7NDstart*_c_***	5/5	5/5	5/5	5/5

*Significant difference compared to wild type determined by Fisher’s exact test (p < 0.05). ND, not determined.

Comp7Nstop*_c_* and Comp7NΔstart*_c_* spirochetes were recovered from heart tissue to the same extent as the wild type and Comp7N*_c_* strains (**Table 2**). Moreover, qPCR analysis of total DNA from the tissues showed no statistically significant differences in spirochete burden between any of the tested strains (*P*<0.05, **Figure 1C**). These combined findings demonstrate that neither introduction of a premature stop codon nor the destruction of the potential start codon affect the functionality of the gene product in the context of heart tissue colonization, further suggesting that the functional gene product encoded by the *bbd07* intergenic region is not a protein, but rather the sRNA SR0726.

### High-Copy Expression of SR0726 Is Deleterious to Murine Host Infection

**Figure 2:**
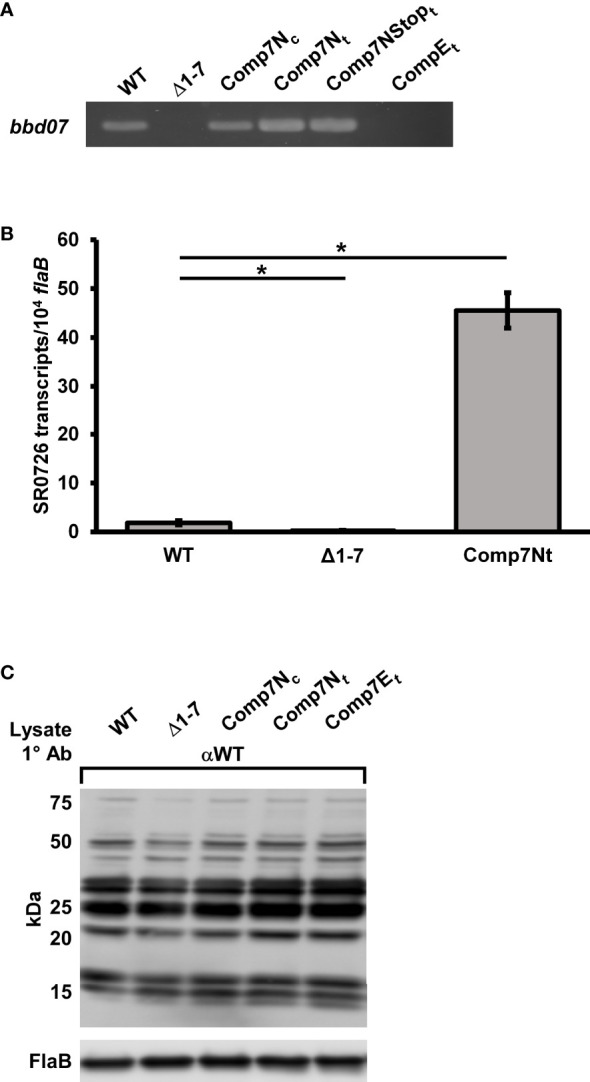
*In trans* complementation of *bbd07* results in elevated SR0726 transcription that does not alter *in vitro* antigen expression. **(A)** PCR verifications of successful *in trans* complementation using P931/P932. **(B)** Assessment of SR0726 transcript levels by indicated strains *in vitro via* qRT-PCR. Triplicate cultures were grown to late log-phase (10^8^ spirochetes mL^-1^). Reactions were performed in duplex with primers for SR0726 (P1001/P1003) and *flab* (P411/P412). Data bars indicate the average number of SR0726 copies per 10^4^ *flaB* copies. Data from negative control reactions using Δ1-7 cDNA is included to illustrate background signal in the assay. Students t-test was performed to assess significance of the differences in SR0726 transcription between each recombinant strain and wild type (*P* < 0.05), and error bars indicate SEM among the biological replicates (n = 3). **(C)** Indicated strains were grown *in vitro* in triplicate under the same conditions used for *in vitro* SR0726 transcript quantification. Bacterial lysates (10^9^ cells per lane) were Western blotted with serum collected from C3H mice infected with wild type for 28 days. FlaB protein from these lysates was probed with polyclonal anti-FlaB antibodies as a loading control. Approximate molecular weights are indicated to the left. Asterisk indicates significant difference in SR0726 transcription levels between indicated strains by Students t-test.

Our laboratory recently reported that absence of the region encoding SR0726 affects antigen expression *in vivo*, which could potentially explain the observed attenuation in tissue colonization and pathogenesis by the mutant (Casselli et al., 2019). Thus, it was hypothesized that SR0726 overexpression may lead to altered murine infectivity due to abnormal antigen production. *B. burgdorferi* has been shown to maintain the pBSV2G shuttle vector at a higher copy number than its native plasmids, and expression levels of genes complemented on this vector can be elevated compared to wild type (Elias et al., 2003; Tilly et al., 2006). Thus, *in trans* complemented strains that harbored *bbd07* gene copies on pBSV2G were generated in the Δ1-7 mutant background to assess the effects of SR0726 overexpression. Complementation was performed by transforming Δ1-7 cells with pBSV2G carrying either a wild-type copy of *bbd07* (Comp7N*_t_*) or a copy containing an early stop codon (Comp7Nstop*_t_*). An empty vector control strain harboring pBSV2G without a *bbd07* copy was also generated (CompE*_t_*). PCR verification of the presence or absence of *bbd07* DNA in these strains is illustrated in **Figure 2A**, and isogenecity to the parent strain was confirmed *via* multiplex PCR (data not shown) (Bunikis et al., 2011).

To determine the relative *in vitro* expression levels of SR0726 in the wild type and Comp7N*_t_*, qRT-PCR analysis was performed using cDNA derived from pooled triplicate cultures of wild type, Δ1-7, and Comp7N*_t_* grown to late log-phase (1x10^8^ cells mL^-1^). As shown in **Figure 2B**, a ~24-fold increase in SR0726 transcription by Comp7N*_t_* spirochetes compared to the wild type was observed. Wild-type transcription of SR0726 was found to be low, producing less than two SR0726 copies per 10^4^ *flaB* copies. These results indicate that transcriptional induction of SR0726 may be tightly regulated, and that residence of the encoding sequence on a high-copy vector results in elevated *in vitro* transcription.

To assess the effects of high copy SR0726 expression on murine infection, groups of five C3H mice each were needle inoculated (5x10^3^ total spirochetes) with wild type, Comp7N*_t_*, Comp7Nstop*_t_*, or CompE*_t_* spirochetes. Infection was monitored weekly by culturing of tissues as before. Interestingly, while infection was established in all mice inoculated with wild type or the CompE*_t_* control clone, infection was not established in any of the mice inoculated with Comp7N*_t_* or Comp7Nstop*_t_* spirochetes (**Table 3**). To ensure reproducibility, a second independent infection assay with the Comp7N*_t_* and Comp7Nstop*_t_* clones was conducted with the same results. Next, groups of three C3H mice each were inoculated with 10^4^, 10^5^, or 10^6^ Comp7N*_t_* spirochetes alongside three mice infected with 10^4^ wild type spirochetes in order to test if the infectivity defect associated with SR0726 overexpression could be overcome by increased inoculum dosage. While all wild type-inoculated mice became infected, none of the mice inoculated with Comp7N*_t_* became infected, indicating that this phenotype is not dose dependent (data not shown). To determine whether the lack of infectivity exhibited by these strains in C3H mice was due to an inability to evade the adaptive immune response, Comp7N*_t_* and Comp7Nstop*_t_* spirochetes were used to inoculate groups of five immunodeficient SCID mice. Neither strain was able to establish infection in any of the mice tested during the 28-day experiment (**Table 4**). Together, these findings suggest that high-copy expression of SR0726 renders spirochetes unable to establish murine infection, even in the absence of an adaptive immune response.

**Table 3:** Infectivity of *in trans* complemented strains in C3H mice.

Strain	Timepoint/tissue			
	Day 7/blood	Day 14/ear	Day 21/ear	Day 28/ear
**WT**	5/5	5/5	5/5	5/5
**Δ1-7**	13/13	ND	ND	6/13*
**Comp7N_c_**	5/5	5/5	5/5	5/5
**Comp7N*_t_***	0/10*^#^	0/10*^#^	0/10*^#^	0/10*^#^
**Comp7Nstop*_t_***	0/10*^#^	0/10*^#^	0/10*^#^	0/10*^#^
**CompE*_t_***	5/5	5/5	5/5	5/5

*Significant difference compared to wild type determined by Fisher’s exact test (p < 0.05). ND, not determined.

^#^Combined data from two independent experiments with n=5.

**Table 4:** Infectivity of *in trans* complemented strains in SCID mice.

Strain	Timepoint/tissue			
	Day 7/blood	Day 14/ear	Day 21/ear	Day 28/ear
**WT**	5/5	5/5	5/5	5/5
**Δ1-7**	5/5	5/5	5/5	5/5
**Comp7N*_t_***	0/5*	0/5*	0/5*	0/5*
**Comp7Nstop*_t_***	0/5*	0/5*	0/5*	0/5*

*Significant difference compared to wild type determined by Fisher’s exact test (p < 0.05). ND, not determined.

### High-Copy Expression of SR0726 Does Not Alter Antigen Expression *In Vitro*

Recently, we showed that the murine tissue colonization defect and attenuated pathogenesis exhibited by the Δ1-7 mutant during murine infection was associated with dysregulated antigen expression (Casselli et al., 2019). Considering this, along with the finding that the *in trans bbd07* complement strains were non-infectious in mice, it seemed possible that overexpression of SR0726 might also result in dysregulated antigen expression that in turn renders those strains non-infectious. Because SR0726 transcription by Comp7N*_t_* spirochetes is highly elevated during *in vitro* cultivation (**Figure 2B**), we predicted that any potential resultant protein expression changes would be observable under the same conditions.

To assess the effects of SR0726 overexpression on overall antigen production, cultures of wild type, Δ1-7, Comp7N*_c_*, Comp7N*_t_*, and CompE*_t_* spirochetes were grown in triplicate under the same conditions used to quantify SR0726 transcript levels in the Comp7N*_t_* strain. Pooled protein lysates (10^9^ total cells) of each strain were subjected to Western blot analysis using murine immune sera harvested from mice that had been infected with wild-type *B. burgdorferi* for 28 days. Unexpectedly, no notable differences were observed between the *in vitro* antigenic profiles of the tested strains (**Figure 2C**). This result suggests that the non-infectious phenotype exhibited by Comp7N*_t_* is unlikely to be due to dysregulated antigen expression during inoculum preparation of *in vitro*-grown spirochetes. Western blotting of *in vitro*-grown wild-type whole cell lysates using Comp7N*_t_* antisera was also attempted, but it was found that insufficient antibody titers are generated following inoculation with this non-infectious clone.

### Expression of SR0726 Is Induced During Murine Infection

**Figure 3:**
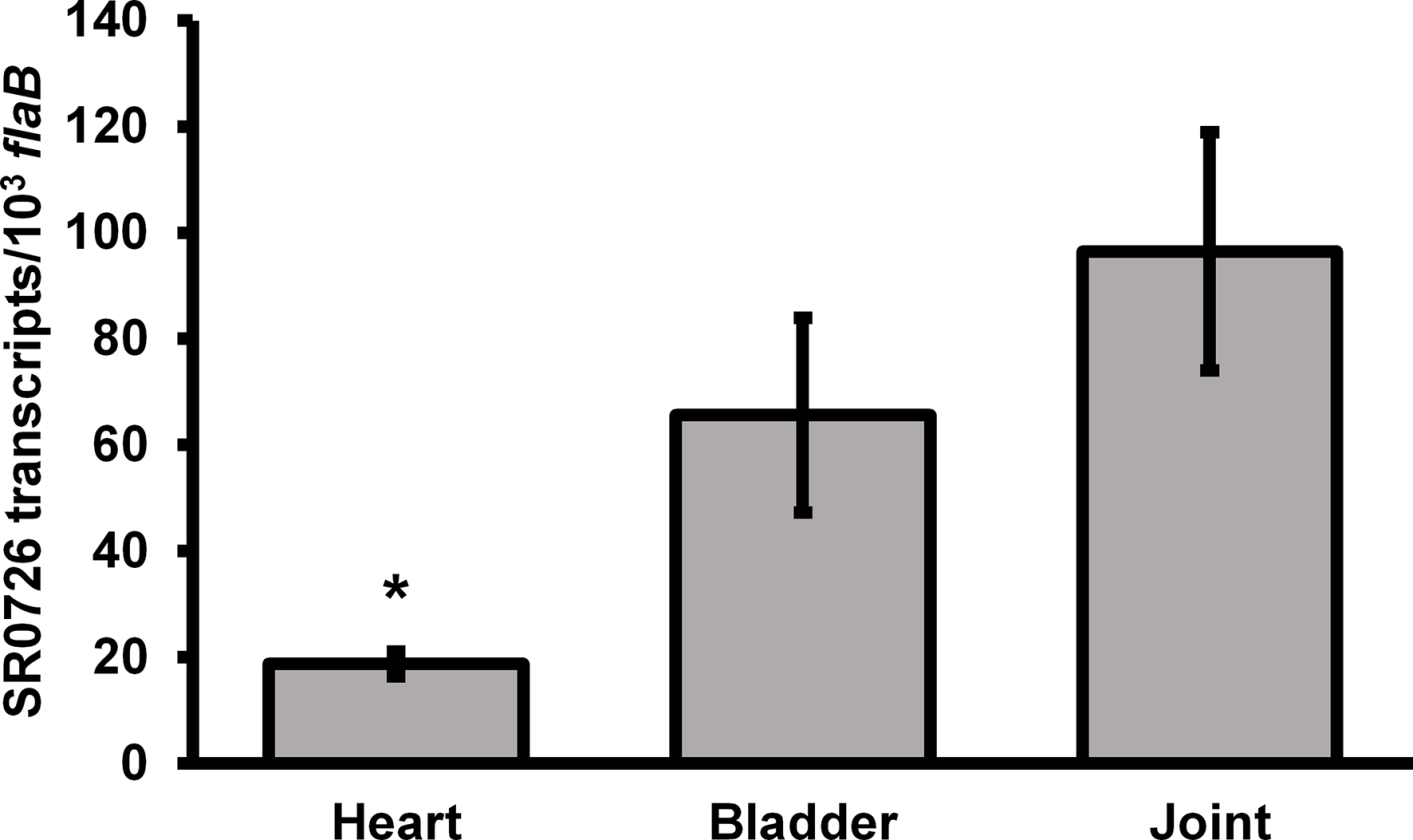
SR0726 transcription is induced during mammalian infection. Three mice were inoculated subcutaneously with 10^5^ wild type spirochetes. Infection was verified as before. Tissues were harvested for RNA extraction on day 28 post-infection. cDNA derived from indicated tissues was used in qRT PCR reactions similar to those used for assessment of SR0726 transcript levels *in vitro*. Error bars indicate SEM among biological replicates (n = 3). Asterisk indicates statistically different group (*P* < 0.05) as determined by one‐way ANOVA followed by all pairwise multiple comparison (Holm‐Sidak).

Transcription of the SR0726 locus was previously shown to increase during *in vitro* growth at 37°C compared to 23°C, suggesting that it may be upregulated during transmission and/or infection of the mammalian host (Popitsch et al., 2017). To determine whether SR0726 transcription is in fact induced in the mammalian host, and to assess any potential expression differences in spirochetes colonizing various tissue sites, SR0726 transcripts were quantified from infected murine tissues by qRT-PCR. Groups of three mice each were infected with 1x10^5^ total wild-type spirochetes, and heart, bladder, and joint tissues were harvested at 28 days post infection for total RNA extraction and subsequent analysis. Relative abundance of SR0726 expression was calculated for each tissue. Expression of SR0726 was detected in each tissue examined, with an average expression level of approximately 20, 65, and 95 copies of SR0726 per 10^3^ copies of *flab* in spirochetes colonizing either the heart, bladder, or joints, respectively (**Figure 3**). These values range from a 100- to 400-fold increase in SR0726 transcript levels relative to that observed for *in vitro*-grown wild type spirochetes (~2 copies of SR0726 per 10^4^ copies of *flab*, see **Figure 2B**), supporting the hypothesis that SR0726 transcription is upregulated during mammalian infection. Despite SR0726 transcription being ~100-fold higher in heart-resident spirochetes than those grown *in vitro*, this *in vivo* SR0726 expression level was significantly less than that observed for spirochetes colonizing joint tissue (*P<*0.05). Differences in SR0726 transcription between heart and bladder, and bladder and joint did not reach significance.

### Targeted Deletion of SR0726 Results in an Altered Transcriptome

**Figure 4:**
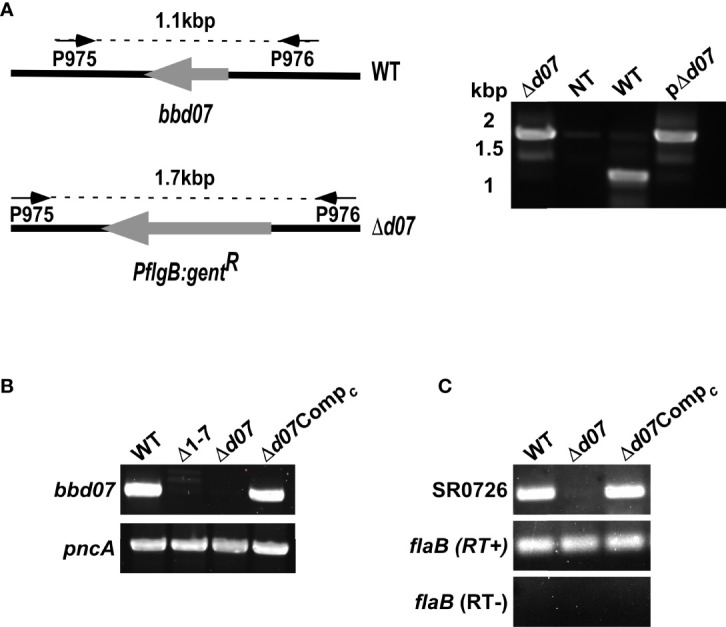
Targeted deletion of *bbd07*. **(A)** Schematic illustrating the relevant segment of lp17 in the wild type (WT) and the *bbd07* knockout (Δ*d07*). Screening primer annealing sites and expected amplicon lengths are shown above each schematic with arrows connected by dashed lines. **(B)** PCR confirmations of presence or absence of SR0726 genetic region. **(C)** Verification of presence or absence of SR0726 transcription in indicated strains by RT-PCR using P931/P932.

To further examine a potential regulatory role for SR0726, we sought to determine if deletion of SR0726 affects the transcriptome of *B. burgdorferi*. To eliminate any possible secondary effects brought about by the additional loss of *bbd01-bbd06* in the Δ1-7 mutant clone, it was necessary to generate a knockout clone that lacked only the *bbd07* locus. Deletion of the 317 bp region of lp17 encoding SR0726 was performed through allelic exchange using a suicide vector-derived plasmid construct containing a gentamycin resistance cassette flanked by upstream and downstream regions of DNA homologous to lp17 (**Figure 4A**). A positive clone (Δ*d07*) was selected and recovered *via* PCR screening as shown in **Figure 4B**. The endogenous plasmid profile of this clone was identical to the wild type 5A4 parent strain with the exception of cp32-6, which is not required for infectivity (Brisson et al., 2013). Deletion was further verified by RT-PCR to ensure the absence of SR0726 transcription (**Figure 4C**). This clone did not display any apparent defects in *in vitro* growth rate, morphology or motility, consistent with the absence of these defects in the Δ1-7 mutant tested in a previous work (Casselli et al., 2019). Numerous attempts to complement the SR0726 region *via* allelic exchange were unsuccessful. As an alternative strategy, a complement clone was generated by transforming Δ*d07* cells with gDNA from a previously described strain harboring an lp17 plasmid in which the *bbd01-bbd03* region was replaced with a kanamycin resistance gene (Δ*d07*Comp*_c_*)*. bbd01-bbd03* are known to be dispensable in terms of murine tissue colonization (Casselli et al., 2019). This plasmid replacement approach has been utilized in several past studies where traditional complementation was not achievable in other *B. burgdorferi* mutant clones (Imai et al., 2013; Raffel et al., 2014). This clone was PCR negative for a gentamycin resistance cassette and did not grow in culture in the presence of gentamycin, which confirmed complete plasmid replacement (data not shown).

**Figure 5:**
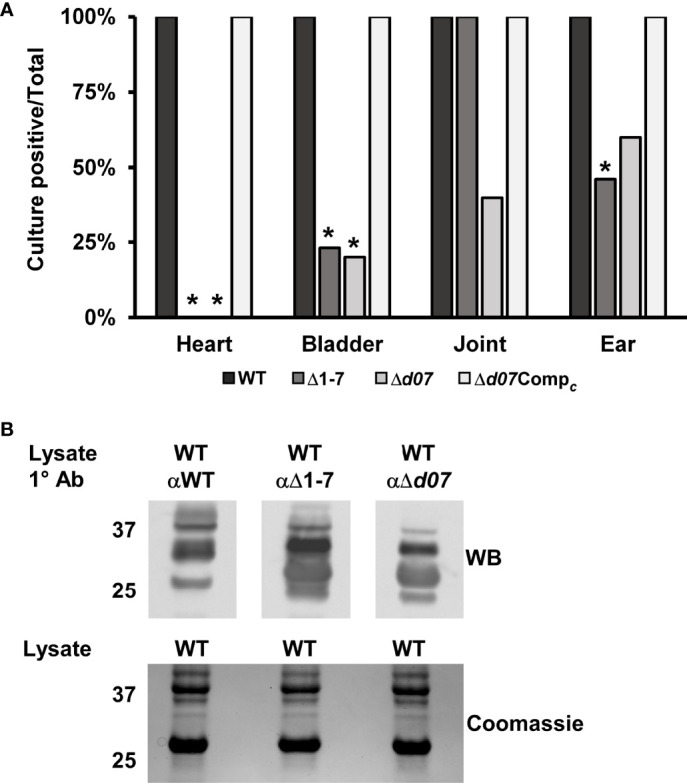
Targeted deletion of *bbd07* results in a phenotype comparable to Δ1-7. **(A)** Groups of 10, 5, and 4 mice were infected with 5x10^3^ spirochetes of the indicated strains, respectively. Indicated tissues were harvested at day 28 post-infection and cultured in BSK for detection of spirochete growth as described above. Data bars indicate the percentage of mice in each group that were culture positive for spirochete growth in the indicated tissues. Δ1-7 data was transposed from (Casselli et al., 2019). Asterisks indicate statistically significant difference compared with wild type as determined by Fisher’s exact test (*P* < 0.05). **(B)** Anti-sera were extracted from mice (n = 5) infected with the indicated strains for 28 days. The sera were used as the primary antibody in a western blot probing protein lysate of *in vitro* cultivated wild type spirochetes. A gel loaded identically to that used for the Western blot was Coomassie stained to serve as a protein loading control and is shown below the blot.

A mouse infection assay was carried out to ensure the newly generated Δ*d07* mutant strain would exhibit an *in vivo* phenotype similar to the Δ1-7 mutant clone. Groups of five C3H mice each were needle inoculated with the wild type, Δ*d07*, or Δ*d07*Comp*_c_* at a dose of 5x10^3^ total spirochetes. All mice became infected as determined by culture positivity of blood and ear tissue collected weekly following infection. At day 28 post infection, heart, bladder, joint, and ear tissue were harvested and cultured for detection of spirochete growth by dark field microscopy as described before. As was previously observed for Δ1-7, none of the heart tissue cultures from Δ*d07*-infected mice were positive for spirochete growth, further demonstrating that SR0726 transcription is required for heart tissue colonization in mice (**Figure 5A**). Unexpectedly, joint tissue colonization was attenuated for Δ*d07* compared to wild type, with spirochetes being recovered from that tissue in only 2/5 Δ*d07*-infected mice. This contrasts with Δ1-7, which was recovered from joints of all 13 mice at the 28-day time point, despite its recovery from joints being attenuated at day 14 in prior experiments (Casselli et al., 2019). Δ*d07*Comp*_c_* spirochetes were recovered from all tissues in all mice, supporting the validity of the observed phenotype of targeted *bbd07* deletion.

As a final confirmation that the Δ*d07* mutant exhibits similar defects to those of the Δ1-7 strain, the *in vivo* antigenic profile of Δ*d07* was compared to those of the wild type and Δ1-7 strains. This was done by subjecting *in vitro*-grown wild-type spirochetes to Western blot analysis using pooled antisera from groups of 3 mice infected for 28 days with either wild-type, Δ1-7, or Δ*d07* spirochetes. Sera from Δ*d07*- and Δ1-7-infected mice displayed detectable reactivity to at least two additional antigens compared to sera from wild type-infected mice, indicating altered expression of these antigens during murine infection by strains lacking SR0726 transcription (**Figure 5B**).

The Δ*d07* mutant was then analyzed by RNA-seq to determine if the absence of SR0726 transcription affects the *B. burgdorferi* transcriptome. To accomplish this, triplicate cultures of wild-type or Δ*d07* spirochetes were grown to a density of 1x10^8^ spirochetes mL^-1^ at 37°C following a temperature shift involving a 1:100 subculture from room temperature cultures. This temperature shift was performed in an attempt to induce SR0726 expression by the wild type (Popitsch et al., 2017). Pooled RNA from each strain was DNase treated and sent to the Novogene Corporation for library preparation, sequencing, and data analysis. **Table 5** shows a list of 17 transcripts that were differentially expressed between wild type and Δ*d07* transcriptomes with a log_2_-fold change > ± 2. In total, 91 transcripts were differentially detected with a log_2_-fold change > ± 1, omitting those transcripts encoded by cp32-6, which is absent in the Δ*d07* clone. Full DEG analysis results can be found in **Supplementary Tables 2, 3**.

**Table 5:** Genes differentially expressed by Δd07 compared to wild *type in vitro*.

Gene ID	Gene Description	Read count WT	Read count Δ*d07*	Log_2_ fold change^a^	P-value
**BB_0360**	Hypothetical protein	1.341	45.631	5.0879	3.51E-12
**BB_0044**	Hypothetical protein	5.664	38.242	2.7551	2.56E-07
**BB_0234**	Membrane protein	34.734	157.065	2.1769	8.32E-20
**BB_0469**	Signal peptidase II	20.945	86.271	2.0423	9.18E-11
**BB_0157**	Hypothetical protein	81.618	333.602	2.0312	6.09E-37
**BB_0759** ^b^	DUF368 domain-containing protein	18.038	4.293	-2.0708	0.0024284
**BB_I21**	ParA family protein	142.739	31.852	-2.1639	1.82E-18
**BB_0206**	RsmD	315.890	68.397	-2.2074	8.25E-40
**BB_0733**	PlzA	922.850	194.709	-2.2448	4.17E-115
**BB_A57**	Surface lipoprotein	43.306	8.287	-2.3855	3.05E-07
**BB_0365**	Periplasmic lipoprotein	2919.265	521.621	-2.4845	0
**BB_K50**	Immunogenic protein P37	337.059	57.414	-2.5535	7.30E-50
**BB_0760**	Nucleoside triphosphate pyrophosphohydrolase family protein	114.042	18.172	-2.6497	1.24E-18
**BB_K49**	Hypothetical protein	119.409	18.871	-2.6616	1.60E-19
**BB_A69**	Complement regulator-acquiring protein	570.361	82.676	-2.7863	5.12E-91
**BB_0046**	Ribonuclease HII	146.913	21.168	-2.795	8.35E-25
**BB_K47**	Hypothetical protein	492.619	70.894	-2.7967	3.67E-79

a Genes that displayed a log_2_-fold change at least of ±2 in transcription between the strains are listed.

b Bold line indicates separation of positive and negative Log_2_ fold change values.

**Figure 6:**
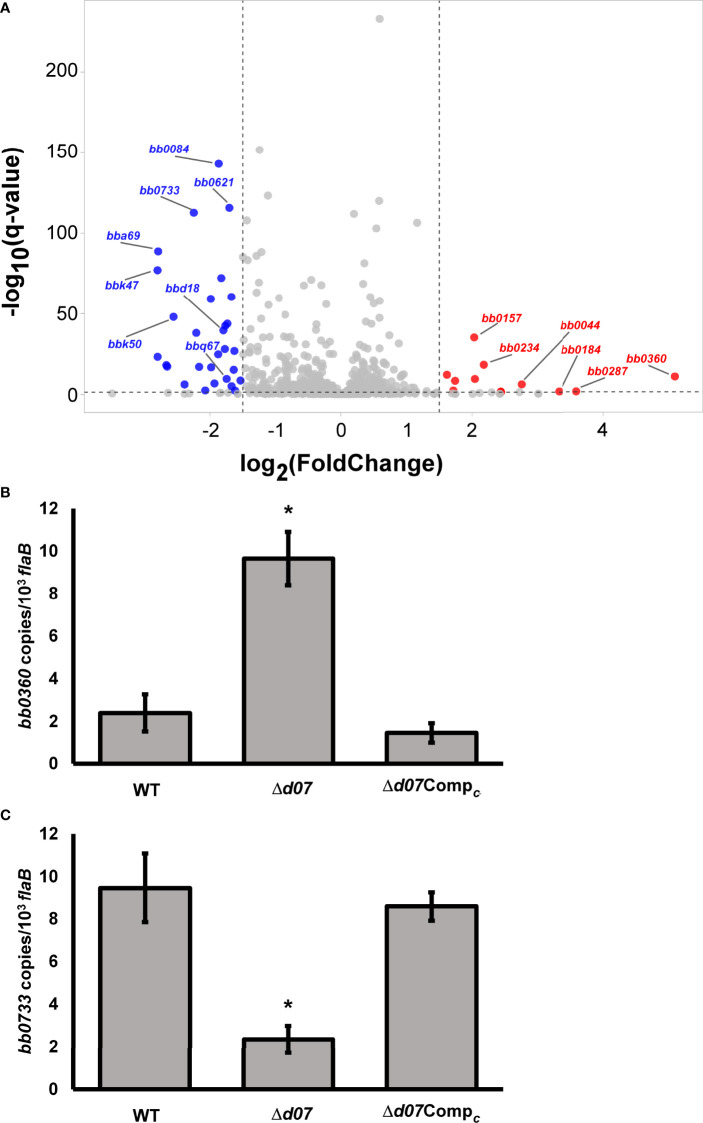
*bbd07* deletion results in an altered *in vitro* transcriptome. **(A)** Volcano plot illustrating differentially expressed genes (DEGs) following targeted deletion of *bbd07.* DEGs with a positive Log_2_ fold change were expressed at a greater level in Δ*d07* than in wild type and are shown as red dots. DEGs with negative Log_2_ fold change were expressed at lower level in Δ*d07* than in wild type and are shown as blue dots. Red and blue dots correspond to genes with log_2_-fold change in expression of ±1.5, and with a -Log_10_ (qvalue) greater than 1.5. **(B, C)** qRT-PCR analysis comparing relative transcription of *bb0360* **(B)** and *bb0733* **(C)** between indicated strains. Each bar represents the average value from three unpooled biological replicates (cultures) per strain, with three technical replicates performed for each biological replicate. Error bars indicate SEM among biological replicates (n = 3). Asterisk indicates statistically different group (*P* < 0.05) as determined by one‐way ANOVA followed by all pairwise multiple comparison (Holm‐Sidak).

The most significant transcriptional difference observed was in *bb0360*, which was detected approximately 34-fold more from Δ*d07* compared to wild type (*P*=3.51x10^-12^) and encodes a hypothetical protein. Sultan et al. demonstrated that this gene is the first in a co-transcribed operon including the subsequent four genes (*bb0361-bb0364*) (Sultan et al., 2010). Despite this, none of the other transcripts in the putative polycistron were detected differentially between the strains tested. *bb0733* was detected 5.5-fold less from Δ*d07* compared to wild type and encodes PlzA, a well characterized c-di-GMP binding protein. A volcano plot illustrating a compilation of the differential expression analysis is shown in **Figure 6A**. In order to corroborate the findings of this experiment, qRT-PCR analysis was performed to compare the relative transcription levels of *bb0360* and *bb0733* between wild type, Δ*d07*, and Δ*d07*Comp*_c_*. Consistent with the trends observed by RNAseq, *bb0360* transcription was significantly elevated (*P*<0.05, **Figure 6B**) and *bb0733* transcription significantly diminished (*P*<0.05, **Figure 6C**), in Δ*d07* compared to wild type and Δ*d07*Comp*_c_*. The fold change in *bb0733* transcription between wild type and Δ*d07* was nearly identical to what was observed by RNAseq, while the degree of difference in *bb0360* transcription was not as stark. Combined, this data demonstrates that SR0726 has effects on the *in vitro* transcriptome of *B. burgdorferi*, and that the overall effects of its activity may be diverse.

### SR0726 May Be Post-Transcriptionally Processed Into a Mature sRNA With Complementarity to *bb0360*

**Figure 7:**
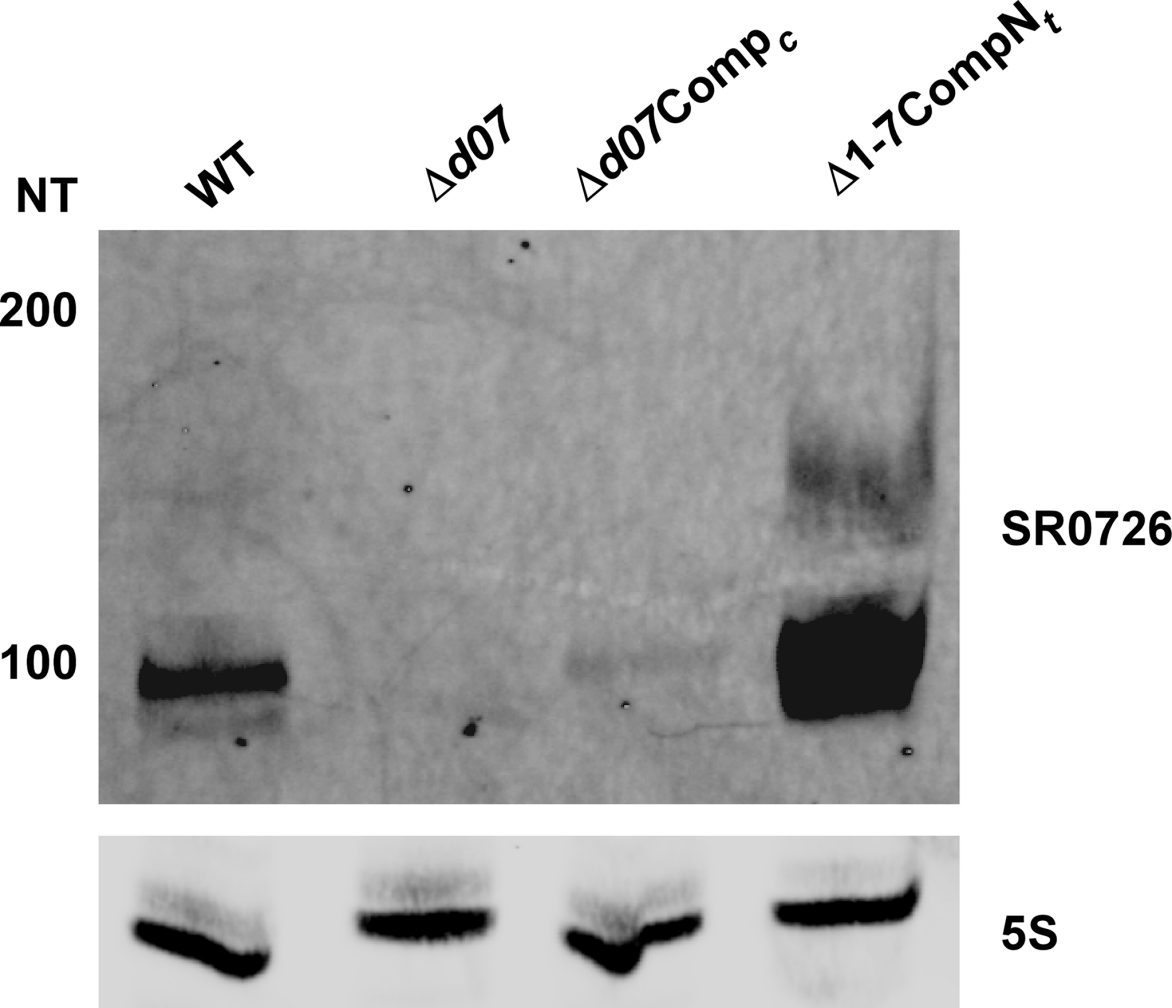
Confirmation of SR0726 transcript size. Northern blot of total RNA extracted from *in vitro* grown wild type, Δ*d07*, Δ*d07*Comp*_c_*, and SR0726 overexpression strain (CompN*_t_*). 5S rRNA serves as the loading control for each strain.

Sequencing of the temperature-dependent sRNA transcriptome by Popitsch and colleagues has previously revealed that SR0726 is likely expressed as a longer proto-transcript which is processed into a 98 nucleotide (nt) sRNA corresponding to bps 4269-4366 of lp17 that is highly favored during growth *in vitro* at 37°C compared to at room temperature (Popitsch et al., 2017). To test this potentiality and to confirm the existence of a stably transcribed SR0726 sRNA, Northern blot analysis was performed using a probe complementary to a run of nucleotides contained in the sequence reported in the study mentioned above. RNA from wild type, Δ*d07*, Δ*d07*Comp*_c_*, and Comp7N*_t_* was prepared identically to that used for the RNAseq analysis reported above. Comp7N*_t_*-derived RNA was used in this experiment to ensure detectability of the transcript, as this clone had been shown to overexpress SR0726 (**Figure 2B**). As illustrated by the most intense band in **Figure 7**, this analysis confirmed the presence of a stably transcribed sRNA of approximately 98 nucleotides being produced in each strain except the Δ*d07* mutant. Additionally, a band corresponding to a larger transcript is visible in the wild type and Comp7N*_t_* lanes which may represent the hypothetical unprocessed transcript. The band intensity from Δ*d07*Comp*_c_* RNA was remarkably low and may have been due to variability of SR0726 expression levels *in vitro*.

**Figure 8:**
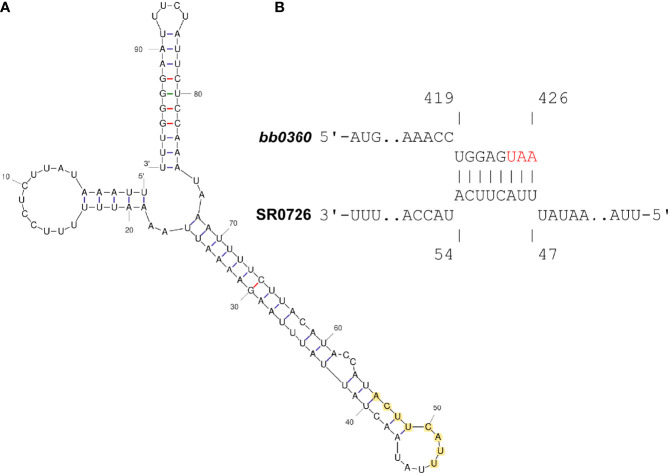
*In silico* analysis predicts potential interaction between SR0726 and 3’ terminus of *bb0360* mRNA. **(A)** Predicted secondary structure of putative SR0726 RNA generated in mfold. **(B)** Potential interaction of SR0726 (lower) within the coding sequence of *bb0360* (upper) generated in intaRNA. Red letters indicate the *bb0360* stop codon. Nucleotides predicted to participate in the interaction are highlighted in yellow on the predicted SR0726 structure.

Using this dominant 98 nt sequence as an input, a folding prediction with the mfold webtool was conducted, and a binding interaction prediction with the *bb0360* coding sequence with intaRNA (Freiburg RNA Tools) was then performed (Zuker, 2003; Busch et al., 2008). These *in silico* analyses revealed a potentially highly structured SR0726 RNA (**Figure 8A**) that may bind through complementarity to the extreme 3’ end of the *bb0360* mRNA (**Figure 8B**).

## Discussion

In this study, we attempted to further characterize the *bbd07* intergenic region of lp17 by determining if the functional product encoded is an sRNA, and by assessing any potential regulatory effects resulting from its targeted deletion. Our findings show that genetically complemented strains of the Δ1-7 mutant featuring mutations aimed at eliminating potential protein translation (Comp7stop*_c_* and Comp7Δstart*_c_*) colonized heart tissue at levels comparable to the wild type and native *in cis* complement (Comp7N*_c_*). This evidence, along with the ability to detect a stable transcript by Northern blot, strongly suggests that this genetic locus encodes sRNA SR0726, and that the lack of production of this sRNA in our mutant clones is responsible for their observed phenotypes. While we cannot entirely rule out the possibility that a small peptide is translated from this genetic region, our data show that the phenotype associated with deletion of this sequence is not due to the absence of a putative BBD07 protein product. Proceeding under the notion that the functional product encoded by this region is a regulatory sRNA, we reasoned that it may have drastic effects on multiple bacterial characteristics if it were transcribed from a high-copy plasmid, and that investigation of these effects could provide insight into the function of SR0726. Indeed, spirochetes overexpressing SR0726 were unable to establish infection in C3H mice using an inoculum of up to 10^6^ spirochetes and were also found to be non-infectious in SCID mice. This is a uniquely severe phenotype, as many sRNAs of other of pathogenic bacteria have been observed to carry subtle deletion or overexpression phenotypes, due in part to functional redundancy and incomplete inhibition/activation of target mRNA expression (Beisel and Storz, 2010; Storz et al., 2011; Caldelari et al., 2013; Barquist and Vogel, 2015; Lybecker and Samuels, 2017). The severity of phenotypes associated with *bbd07* deletion and SR0726 overexpression suggest that the sRNA may serve a key regulatory function critical for mammalian infection. If so, it may either have robust regulatory activities on multiple targets that together confer a fitness advantage and tissue colonization capacity, or it may regulate a single target early in a regulatory pathway, causing multiple and critical downstream effects. In either case, it appears that the effects of the absence or overexpression of SR0726 cannot be fully overcome *in vivo* by compensatory or redundant regulatory mechanisms. Another possible explanation for the severe phenotype resulting from SR0726 overexpression is that an excess of the transcript may lead to the sequestration of RNA binding proteins such as Hfq*_Bb_*, which may disrupt various other cellular functions, that leads to the lack of infectivity (Lybecker et al., 2010). Experiments are currently underway to test these mechanistic possibilities.

Recently, we reported that the Δ1-7 mutant displays a dysregulated antigenic proteome during murine infection (Casselli et al., 2019). Specifically, two yet unidentified proteins appeared to be de-repressed in the absence of SR0726 transcription. Based on this, the lack of infectivity exhibited by Comp7N*_t_* seemed likely to be the result of antigen dysregulation during *in vitro* growth and inoculum preparation, and perhaps over-repression of protein(s) required for infection in the pre inoculum. Unexpectedly, however, SR0726 overexpression (Comp7N*_t_*) appeared to have no effect on antigen expression *in vitro* despite changes being observed in preliminary iterations of the experiment. One possible explanation for this outcome is that the lack of infectivity exhibited by Comp7N*_t_* spirochetes is not due to altered antigen expression, but rather in the expression of cytosolic proteins. Alternatively, it may be that altered antigen expression is only detectable in spirochetes when propagating within the infected host environment. Indeed, we observed SR0726 to be transcribed 20-fold higher by wild type spirochetes colonizing heart tissue compared to *in vitro*-grown spirochetes. Even higher transcription levels were detected in bladder and joint tissues. This observation points to the importance of the timing and regulation of SR0726 transcription, that is, overexpression in culture prior to inoculation mitigates infectivity (Comp7N*_t_*), while induction to even higher levels *in vivo* is critical for pathogenesis. While previous studies have found SR0726 transcription to be RpoS- and temperature-dependent, it seems highly worthwhile to more precisely define which conditions and/or regulatory elements promote this induction, and when the induction occurs within the enzootic cycle (Boardman, 2008; Ouyang et al., 2008; Popitsch et al., 2017; Drecktrah et al., 2018).

Targeted deletion of *bbd07-* the 317 bp intergenic region of lp17 encoding SR0726- resulted in an infectious phenotype similar to the previously characterized Δ1-7 mutant clone (Casselli et al., 2019). Most importantly, Δ*d07* spirochetes were not capable of murine heart tissue colonization. The altered *in vivo* antigenic profile of the Δ1-7 mutant demonstrated in a previous study was also reproduced in the current work with the newly generated Δ*d07* mutant. Global transcriptomic analysis comparing wild type and Δ*d07* revealed potential insight into the severe phenotypes associated with SR0726 absence and overexpression. Specifically, *bb0360* was found to be approximately 34-fold more abundant in spirochetes lacking SR0726. While this gene is annotated as a hypothetical protein with no predicted function, it has been shown to be co-transcribed with four other genes (*bb0360-bb0364*). In that study, *bb0363* was revealed to function as a cyclic-di-GMP phosphodiesterase required for optimal motility and infectivity (Sultan et al., 2010). *bb0360* was the only transcript of this polycistron that was differentially detected in our experiment. Using the 98 nt dominant sRNA sequence reported by Popitsch and colleagues and corroborated by Northern blot data reported herein, *in silico* analyses were performed and predicted a highly structured SR0726 RNA which may bind through complementarity to the extreme 3’ end of the *bb0360* mRNA. Interestingly, the region of predicted binding contains the Shine-Delgarno sequence for the CDS of the downstream gene *bb0361*, which encodes a predicted ATP-binding protein (Fraser, 1997; Galperin et al., 2001).

Transcription of *bb0733* was also differentially detected (5.5-fold less in Δd07) from RNAseq analysis, which encodes a known c-di-GMP binding protein PlzA (Pitzer et al., 2011). In *B. burgdorferi*, c-di-GMP serves as a cyclic nucleotide second messenger that promotes expression of tick phase-specific genes through activation of the HK1-Rrp1 regulon, which is critical for spirochete survival in ticks (Novak et al., 2014). More recently, PlzA was shown to contribute to mammalian host adaptation in the absence of c-di-GMP (Groshong et al., 2021). Considering the combination of previous observations and those reported herein, it appears that the overall outcomes of SR0726 activity are diverse and impactful on the virulence of *B. burgdorferi*. Further work is required to solidify a regulatory connection between SR0726 and these genes, and to clarify the mechanism of any putative regulatory interactions.

To date, lp17 has been a largely understudied component of the *B. burgdorferi* genome. However, recent findings have indicated that the plasmid may serve as a regulatory hub encoding multiple factors, including protein and sRNA molecules that interact with the regulatory mechanisms required for this pathogen to transit through the enzootic cycle (Sarkar et al., 2011; Casselli et al., 2012; Hayes et al., 2014; Medina-Pérez et al., 2020). As techniques for studying sRNA biology in bacteria continue to emerge and evolve, it is likely that further study of lp17-encoded sRNAs will reveal new mechanisms of gene regulation that will expand our understanding of this steadily emerging pathogen. This and other recent studies are elucidating the critical nature of lp17-encoded functionalities to the fitness of the Lyme disease pathogen in each environment it encounters in nature. Further research in this area holds the promise of revealing enigmatic mechanisms of pathogenesis that may serve as points of opportunity for the prevention of the debilitating disease.

## Data Availability Statement

The datasets presented in this study can be found in online repositories. The names of the repository/repositories and accession number(s) can be found in the article/**Supplementary Material**.

## Ethics Statement

The animal study was reviewed and approved by Washington State University Institutional Animal Care and Use Committee.

## Author Contributions

MC and TB designed the experiments. MC performed the experiments. MC and TB analyzed the data and wrote the manuscript. All authors contributed to the article and approved the submitted version.

## Funding

This research was supported by funding to TB through the National Institutes of Health/NIAID grant R21 AI155756.

## Conflict of Interest

The authors declare that the research was conducted in the absence of any commercial or financial relationships that could be construed as a potential conflict of interest.

## Publisher’s Note

All claims expressed in this article are solely those of the authors and do not necessarily represent those of their affiliated organizations, or those of the publisher, the editors and the reviewers. Any product that may be evaluated in this article, or claim that may be made by its manufacturer, is not guaranteed or endorsed by the publisher.
